# Editorial for the Special Issue on Piezoelectric Transducers: Materials, Devices and Applications, Volume II

**DOI:** 10.3390/mi13122192

**Published:** 2022-12-10

**Authors:** Jose Luis Sanchez-Rojas

**Affiliations:** Microsystems, Actuators and Sensors Lab, Institute of Nanotechnology, Universidad de Castilla-La Mancha, 45071 Toledo, Spain; joseluis.saldavero@uclm.es

This is the second volume of the Special Issue focused on piezoelectric transducers, covering a wide range of topics, including the design, fabrication, characterization, packaging, and system integration or final applications of mili/micro/nano-electro–mechanical systems-based transducers, featuring piezoelectric materials and devices. This new volume has compiled a total of 14 papers, with the latest advances in areas such as hysteresis compensation [1,2,3], cylindrical piezoelectric transducers [4], flextensional devices for underwater applications [5], electromechanical transformers [6], actuators for image stabilization [7], electric current sensing [8], rotary and linear ultrasonic motors [9,10], locomotion of miniature robots [11], displacement amplification structures [12] nanoparticles with piezoelectric properties [13], and a review paper on aerospace applications [14].

The works published in this issue reflect the advances in the miniaturization of sensors, actuators, and smart systems that are receiving substantial industrial attention, with a wide variety of transducers commercially available or with high potential to impact emerging markets. Substituting existing products based on bulk materials in fields such as automotive, environment, food, robotics, medicine, biotechnology, communications, internet of things, and related technologies, with reduced size, lower cost, and higher performance, is now possible, with potential for manufacturing using advanced silicon integrated circuits technology or alternative additive techniques from the mili- to the micro-scale.

I want to take this opportunity to thank all the authors for submitting their papers to this Special Issue. I also want to thank all the reviewers for their effort and time in assisting with improving the quality of the submitted papers.

In view of the success reached in the number and quality of papers published, we plan to open a third Volume where we hope to continue with the latest advances in piezoelectric transducers and their trend toward miniaturization, efficiency, and new applications.

## References

[1] Wang W., Wang J., Wang R., Chen Z., Han F., Lu K., Wang C., Xu Z., Ju B. (2021). Modeling and Compensation of Dynamic Hysteresis with Force-Voltage Coupling for Piezoelectric Actuators. Micromachines.

[2] Qin Y., Zhang Y., Duan H., Han J. (2021). High-Bandwidth Hysteresis Compensation of Piezoelectric Actuators via Multilayer Feedforward Neural Network Based Inverse Hysteresis Modeling. Micromachines.

[3] Wang W., Han F., Chen Z., Wang R., Wang C., Lu K., Wang J., Ju B. (2021). Modeling and Compensation for Asymmetrical and Dynamic Hysteresis of Piezoelectric Actuators Using a Dynamic Delay Prandtl–Ishlinskii Model. Micromachines.

[4] Fa L., Kong L., Gong H., Li C., Li L., Guo T., Bai J., Zhao M. (2021). Numerical Simulation and Experimental Verification of Electric–Acoustic Conversion Property of Tangentially Polarized Thin Cylindrical Transducer. Micromachines.

[5] Teng D., Liu X., Gao F. (2021). Effect of Concave Stave on Class I Barrel-Stave Flextensional Transducer. Micromachines.

[6] Rendon-Hernandez A.A., Smith S.E., Halim M.A., Arnold D.P. (2021). Hybrid Piezo/Magnetic Electromechanical Transformer. Micromachines.

[7] Sun M., Feng Y., Wang Y., Huang W., Su S. (2021). Design, Analysis and Experiment of a Bridge-Type Piezoelectric Actuator for Infrared Image Stabilization. Micromachines.

[8] He W. (2021). A High-Resolution Electric Current Sensor Employing a Piezoelectric Drum Transducer. Micromachines.

[9] Yang X., Zhang D., Song R., Yang C., Mu Z. (2021). Development of a Rotary Ultrasonic Motor with Double-Sided Staggered Teeth. Micromachines.

[10] Čeponis A., Mažeika D., Makutėnienė D. (2021). Development of a Novel 2-DOF Rotary–Linear Piezoelectric Actuator Operating under Hybrid Bending–Radial Vibration Mode. Micromachines.

[11] Hernando-García J., García-Caraballo J.L., Ruiz-Díez V., Sánchez-Rojas J.L. (2021). Comparative Study of Traveling and Standing Wave-Based Locomotion of Legged Bidirectional Miniature Piezoelectric Robots. Micromachines.

[12] Li Z., Su Z., Zhao L., Han H., Guo Z., Zhao Y., Sun H. (2021). Design and Locomotion Study of Stick-Slip Piezoelectric Actuator Using Two-Stage Flexible Hinge Structure. Micromachines.

[13] Hwangbo S.A., Choi Y.M., Lee T.G. (2021). Influence of Piezoelectric Properties on the Ultrasonic Dispersion of TiO_2_ Nanoparticles in Aqueous Suspension. Micromachines.

[14] Vo T.V.K., Lubecki T.M., Chow W.T., Gupta A., Li K.H.H. (2021). Large-Scale Piezoelectric-Based Systems for More Electric Aircraft Applications. Micromachines.

